# Cardiotoxic Effects of Acute 4.5 mM Caffeine Exposure in 2-to-3-Day-Old Zebrafish are Attenuated by Tricaine 

**DOI:** 10.17912/micropub.biology.002295

**Published:** 2026-08-07

**Authors:** Autumn E Gray, Catherine E Lipovsky

**Affiliations:** 1 Biology Department, Bradley University, Peoria, IL, United States

## Abstract

A previous study reported that 4 mM caffeine halted cardiac activity in 2-to-3-day-old zebrafish embryos by 72 minutes of exposure, although it remains unclear whether this resulted from a combined effect of caffeine and anesthesia. We demonstrate that 4.5 mM Caffeine significantly alters heart rate and rhythm in 2-to-3-day-old zebrafish embryos with or without tricaine anesthesia but rarely abolishes cardiac activity, even after ~24.5 hours of exposure. Notably, tricaine anesthesia co-administration significantly attenuated caffeine-induced bradycardia at ~2.5 hours and reduced the incidence of arrhythmias at both ~2.5 and ~24.5 hours post-treatment.

**Figure 1. Caffeine impairs cardiac activity at ~2.5 and ~24.5 hpt and the effects are mitigated by co-administration with tricaine anesthesia:** ( **A** - **B** ) Heart rate in beats per minute (BPM) of zebrafish embryos continuously treated beginning at 51.5-52.75 hours post-fertilization (hpf, which is between 2 to 3 days post-fertilization, dpf) with E3 embryo medium only (no anesthesia control), E3 + 0.6 mM Tricaine (anesthesia control), 4.5 mM Caffeine alone, or 4.5 mM Caffeine + 0.6 mM Tricaine and measured at ~2.5 hours post-treatment (hpt, **A** ) and ~24.5 hpt ( **B** ). Heart rate is significantly reduced in all caffeine treatments compared to non-caffeine treatments ( **A** , **B** ) but co-administration of tricaine significantly slows the heart rate decrease at ~2.5 hpt ( **A** ). ( **A** ) and ( **B** ) show mean ± SEM and individual replicates are plotted; n=9 for all treatments. Statistical significance was calculated using a one-way ANOVA followed by a post-hoc Tukey test. NS - not significant; *** *p<0.001* . ( **C** - **E** ) Arrhythmia observations at ~2.5 hpt ( **C** , **E** ) and ~24.5 hpt ( **D** , **E** ) demonstrate that co-administration of tricaine is associated with a significant reduction in the prevalence of developing an arrhythmia in caffeine-treated embryos at ~2.5 hpt (𝝌 ^2^ (1, N = 84 *)* = 4.686, *p* = 0.030) and ~24.5 hpt (𝝌 ^2^ (1, N = 85 *)* = 9.408, *p* = 0.002). ( **C** , **D** ) Percentage of embryos that show any form of arrhythmia at ~2.5 hpt ( **C** ) and ~24.5 hpt ( **D** ) are plotted and the total number of embryos that exhibit an arrhythmia are shown in parentheses. ( **E** ) Table showing the number of samples that exhibited arrhythmias and the nature of the arrhythmias observed at ~2.5 and ~24.5 hpt. For the types of arrhythmias observed, the percentage represents the number of embryos that had the listed arrhythmia over the total number of embryos that experienced an arrhythmia in that treatment condition. Statistical significance was calculated between caffeine-treated groups using a Chi-Square Test of Independence. * *p<0.05* ; ** *p<0.01.*

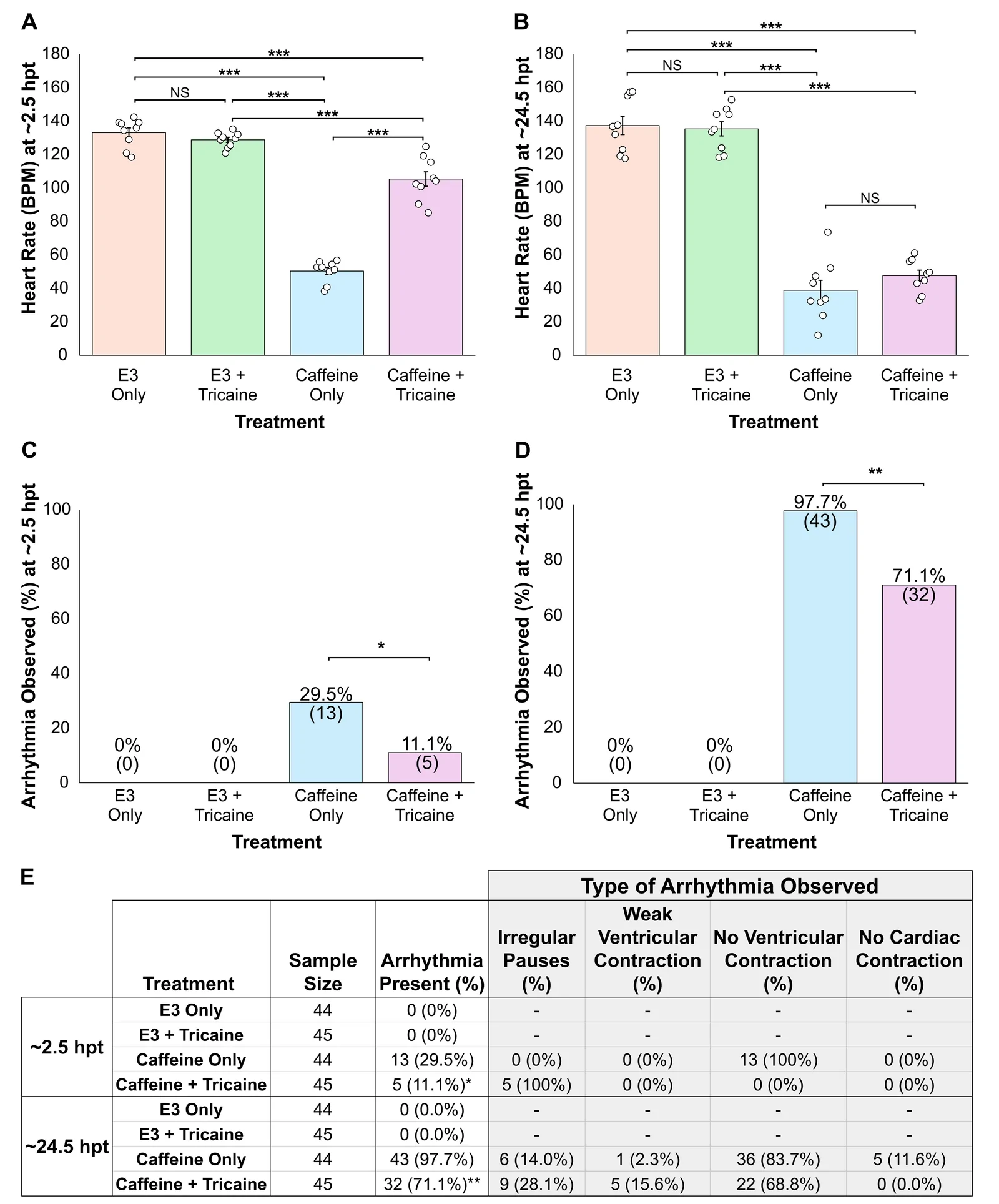



## Description

Caffeine, or 1,3,7-trimethylxanthine, is the most widely consumed psychoactive compound in the world (Nawrot et al., 2003; Heckman et al., 2010). Approximately 89% of the US population is estimated to consume caffeine on a given day, with usual mean intake being 211 mg per day (Fulgoni et al., 2015). Caffeine can cross the placenta, bringing into question how caffeine exposure during pregnancy may affect a developing embryo (Reddy et al., 2024). According to the American College of Obstetricians and Gynecologists (ACOG), pregnant women are advised to consume no more than a moderate level of caffeine (less than 200 mg) per day (ACOG, 2010). Approximately 70% of pregnant women in the United States still consume caffeine during their pregnancy, with reports indicating that some consume even more than the ACOG recommendation (Frary et al., 2005; Weng et al., 2008; CARE Study Group, 2008). This recommendation is based on previous studies that have conflicting results regarding higher levels of caffeine consumption (>200 mg per day), and while these studies examined the risk of miscarriage and intrauterine growth restriction, they did not investigate the effects of caffeine on other important aspects of fetal development, such as cardiovascular function (Mills et al., 1993; Weng et al., 2008; Savitz et al., 2008; CARE Study Group, 2008). 

Zebrafish ( *Danio rerio* ) are a valuable vertebrate model for developmental cardiotoxicity studies because they are externally fertilized, highly fecund, and have optically transparent embryos (Kimmel 1989). Cardiac development proceeds rapidly, with a beating heart tube present by 24 hours post-fertilization (hpf), and functional valves by 48 hpf (Stainier 2001). Zebrafish embryos with disrupted cardiovascular development can survive for approximately one week post-fertilization without a functional heartbeat because their small size allows sufficient oxygen and nutrient delivery through passive diffusion. This feature enables the study of cardiotoxic phenotypes that would otherwise cause early lethality in many other vertebrate model organisms (Stainier and Fishman 1992).

A previous study examining the effect of caffeine on heart rate in zebrafish embryos between 2-to-3 days post-fertilization (dpf) looked at continuous exposure of 1, 4, 8, 10, 12, or 25 mM caffeine and found that heart rate initially decreased and then completely ceased by 197, 72, 44, 20, 16, and 14 minutes after exposure, respectively (Rana et al., 2010). Because these results were obtained in the presence of 0.6 mM tricaine (MS-222) anesthesia to facilitate heart rate measurements by immobilizing the fish, it remains unclear whether this finding was due to a combinatorial effect of caffeine and anesthesia. 

To determine whether anesthesia in combination with caffeine causes the cessation in cardiac activity that has been previously described, we administered 4.5 mM caffeine with or without 0.6 mM tricaine in wild-type zebrafish embryos between 2-3 dpf (~51.25-52.75 hpf). Heart rate and rhythm were recorded at ~2.5 hours post-treatment (hpt). Because cardiac activity persisted at ~2.5 hpt, embryos were maintained in their respective treatments and reassessed at ~24.5 hpt 

At ~2.5 hpt, heart rate did not significantly differ between embryos in the E3 embryo medium Only (133.1 ± 2.8 beats per minute [bpm]) and E3 + Tricaine anesthesia (128.8 ± 1.5 bpm) control conditions ( *p=0.724* ), but it was significantly lower in Caffeine Only (50.4 ± 2.2 bpm; *p<0.001* for all comparisons) and in Caffeine + Tricaine (105.4 ± 4.3 bpm; *p<0.001* for all comparisons), demonstrating 4.5 mM caffeine significantly reduces heart rate ( **Figure 1A** ). Interestingly, the co-administration of tricaine anesthesia significantly attenuated this heart rate decrease, suggesting that tricaine is protective against the caffeine-induced heart rate decrease.

At ~24.5 hpt, similar to ~2.5 hpt, there was no significant difference in heart rate between E3 Only (137.4 ± 5.4 BPM) and E3 + Tricaine (135.4 ± 4.2 BPM) controls ( *p=0.991* ; **Figure 1B** ). As opposed to ~2.5 hpt, heart rate between Caffeine Only (38.9 ± 5.9 BPM) and Caffeine + Tricaine (47.6 ±3.2 BPM) was not significantly different at ~24.5 hpt ( *p=0.577* ). Both caffeine treatments were, however, significantly different from both E3 Only and E3 + Tricaine controls ( *p<0.001* for all comparisons), suggesting that caffeine significantly reduces heart rate at ~24.5 hpt, and the heart rate decrease at this time point is not caused by a combinatorial effect of caffeine and tricaine anesthesia.

During heart rate measurements, heart rhythm was noted. All embryos with cardiac activity had a beating atrium, but ventricular activity was variable. No embryos in E3 Only (0/44, 0.0%) or E3 + Tricaine (0/45, 0.0%) had any arrhythmia at ~2.5 hpt ( **Figure 1C** , **E** ) or ~24.5 hpt ( **Figure 1D** , **E** ). Among caffeine-treated embryos, the incidence of arrhythmia was significantly lower in embryos co-exposed to Caffeine + Tricaine than in embryos exposed to Caffeine Only at both ~2.5 hpt (5/45, 11.1% versus 13/44, 29.5%, respectively; 𝝌 ^2^ (1,N=84)=4.686, *p=0.030* ; **Figure 1C** , **E** ) and ~24.5 hpt (32/45, 71.1% versus 43/44, 97.7%, respectively; 𝝌 ^2^ (1, N=85)=9.408, *p* *= 0.002* ; **Figure 1D** , **E** ). Cessation of cardiac activity was only observed at ~24.5 hpt in a small number of embryos that were treated with caffeine without tricaine (5/44, 11.4%; **Figure 1E** ).

Here, we demonstrate that continuous exposure to 4.5 mM caffeine beginning at 2-3 dpf reduced heart rate and increased arrhythmia incidence in zebrafish embryos, and tricaine significantly attenuated these effects. In contrast to previous reports, complete cardiac arrest was rare, occurring only in a small subset of embryos in the Caffeine Only treatment after ~24.5 hpt (5/44, 11.4%; **Figure 1E** ). Furthermore, among all embryos exhibiting cardiac activity across treatments and timepoints, atrial contractions were consistently present. By ~24.5 hpt, however, ventricular contractions were frequently impaired, with some embryos displaying weak ventricular contractions (1/44 [2.3%] in Caffeine Only; 5/45 [11.1%] in Caffeine + Tricaine), and others exhibiting complete loss of ventricular contraction (36/44 [81.8%] in Caffeine Only; 22/45 [48.9%] in Caffeine + Tricaine; **Figure 1E** ).

Although caffeine is widely recognized as a stimulant, previous studies report conflicting results with respect to caffeine’s effect on heart rate, causing both tachycardia (fast heart rate) and bradycardia (slow heart rate), which may be, in part, due to the dose-dependent differential targets of caffeine (Institute of Medicine [US] Committee on Military Nutrition Research, 2001). Millimolar concentrations of caffeine, such as what was used in the present study, can exert pharmacological effects that include activation of ryanodine receptors (RyRs) on the sarcoplasmic reticulum, which stimulates calcium release from intracellular stores, thereby disrupting intracellular calcium homeostasis and affecting cellular signaling and cardiac rhythm (Porta et al., 2011). Tricaine (MS-222) is a Na ^+^ channel blocker, which means it exerts its anesthetic action by blocking cell membrane excitability and depressing action potential propagation (Frazier and Narahashi, 1975). Based on the individual molecular mechanisms of action of caffeine and tricaine separately, it is possible that the attenuated effect on cardiac activity dysfunction observed when these two are combined may be due to overall reduced excitability of cardiac tissue, however, this was not tested and warrants further study.

Differences between our findings and previous reports may reflect methodological and biological factors. First, the imaging system used in the present study may have enabled detection of weak residual cardiac activity that was below the resolution of earlier studies. Second, embryos in our study were age-matched within a narrow developmental window (51.25-52.75 hpf), whereas previous work examined embryos spanning 48-72 hpf. Given the rapid progression of cardiac development during this period, developmental stage may influence sensitivity to caffeine exposure. A limitation of the present study is that ventricular contractility was assessed qualitatively rather than through quantitative measurements of contractile force, potentially underestimating the prevalence of impaired chamber function. 

Our findings demonstrate that studies investigating the effects of caffeine on cardiac function in zebrafish should carefully consider the potential confounding effects of tricaine anesthesia. Specifically, co-administration of tricaine significantly attenuated several cardiotoxic effects observed following caffeine exposure alone, which may lead to an underestimation of caffeine-induced cardiac dysfunction. Future studies should incorporate quantitative assessments of cardiac function, examine caffeine exposure across precisely defined developmental stages, and investigate the molecular mechanisms through which caffeine and tricaine interact to influence cardiac activity and rhythm.

## Methods

**Zebrafish Husbandry and Maintenance**

Adult wild-type zebrafish ( *Danio rerio* ) were obtained from a local specialty fish store (The Fish Geek, Peoria, IL; https://thefishgeekpeoria.com ). Adult zebrafish were housed and maintained at 28.5°C in a standalone recirculating zebrafish rack (Iwaki Aquatic, LAbREED ITS-Z) and kept on a 12 hour-ON/12-hour OFF light cycle in the animal facility at Bradley University. After breeding, embryos were collected and maintained in 1X E3 embryo medium (5 mM NaCl, 0.17 mM KCl, 0.25 mM CaCl _2_ , 0.16 mM MgSO _4)_ in an incubator at 28.5°C until they were used for experimentation. All procedures were conducted following established protocols and in full compliance with the Bradley University IACUC guidelines.

**Drug Treatments**

Dechorionated zebrafish embryos between 2-3 dpf (~51.5-52.75 hpf) were continuously exposed to one of four conditions: E3 embryo medium alone (no anesthesia control), E3 + 0.6 mM tricaine methanesulfonate (MS-222, Sigma-Aldrich A5040; anesthesia control), 4.5 mM caffeine (Sigma-Aldrich C0750; dissolved in E3), or 4.5 mM caffeine + 0.6 mM tricaine. Two mL of each solution were placed in a well of a 12-well plate. Five embryos were placed in a single well, and each treatment condition had 3 wells per trial. Embryos were kept in an incubator at 28.5°C until heart rates were measured. Anesthesia was deemed effective because all organisms exposed to the 0.6 mM tricaine exhibited loss of a touch response. 

**Heart Rate Analysis**

Heart rate was measured at ~2.5 and ~24.5 hpt. This is because the previous study indicated cardiac activity showed total cessation with a lower concentration of caffeine (4 mM caffeine as opposed to our 4.5 mM) after 72 minutes of continuous caffeine exposure (Rana et al., 2010). Because temperature can affect heart rate, all plates were allowed to come to room temperature (~20.6°C) for at least 25 minutes before heart rates were measured. Heart rate was measured by counting the number of heart beats in 15 seconds and multiplying by 4. In the case where arrhythmias were observed, the number of beats in a full minute were recorded. 

**Heart Rhythm Analysis**

During heart rate measurements the heart rhythm of each embryo was noted. If an arrhythmia was observed, the nature of the arrhythmia was recorded. These arrhythmias included irregular pauses between contractions, only one of two chambers contracting, weak chamber contraction, or absence of any cardiac contraction. “Weak” chamber contraction was defined as a chamber in which the cardiac activity visually appeared as a twitch rather than a full-chamber contraction. All embryos were observed using a Zeiss SV6 microscope on a transmitted light base. 

**Statistical Analysis and Graphing**

For statistical analysis, IBM SPSS Statistics (Version 29.0.2.0 for macOS) was used. For panels ( **A** ) and ( **B** ), a one-way ANOVA followed by multiple comparisons using a post-hoc Tukey test was performed. A *p<0.05* was considered statistically significant. For panels ( **C** - **E** ), a Pearson Chi-Square Test of Independence was performed between caffeine-treated groups at ~2.5 hpt ( **C** , **E** ) and ~24.5 hpt ( **D** , **E** ). A *p<0.05* was considered statistically significant.

Bar graphs were created using Microsoft Excel (Version 16.109.3 for macOS). For panels ( **A** ) and ( **B** ), the bar graphs show the averages of individual wells as individual replicates (n=9 total for each treatment). The heart rate of each individual embryo within a single well was averaged, and each well then served as a replicate. Bars represent the mean of all replicates and error bars indicate standard error of the mean (SEM). For panels ( **C** ) and ( **D** ), the bars represent the percentage of total embryos across all trials (n=42-45 total, depending on the treatment group) in which an arrhythmia was observed.

All data represent three independent experiments (technical replicates) in which at least 12 embryos, from at least three independent matings, were analyzed (biological replicates). Total embryo number for heart rate analysis was n=42-45 for each treatment and varied due to inability to observe the heart beat for a consistent 15 seconds due to movement of the embryo (particularly in treatment groups without anesthesia at ~24.5 hpt) or accidental death from manual handling. Embryos that died due to handling were removed from the analyses. No deaths occurred as a result of the treatments themselves.
